# Healthcare Professionals’ Perceptions of Function-Focused Care Education for Nursing Home Practitioners

**DOI:** 10.3390/ijerph18147587

**Published:** 2021-07-16

**Authors:** Su-Jung Lee, Min-Sun Park, Sung-Ok Chang

**Affiliations:** 1 College of Nursing, Korea University, Seoul 02841, Korea; supercrystal77@daum.net; 2 Department of Nursing, Cheongju University, Cheongju 28503, Korea; minsunpark@cju.ac.kr; 3 College of Nursing, BK21 FOUR R&E Center for Learning Health Systems, Korea University, Seoul 02841, Korea

**Keywords:** function-focused care, shared mental model, nursing home, team-based learning, qualitative method

## Abstract

A nursing home (NH) care environment necessitates a shared cognition-based education model that maintains effective function-focused care (FFC). This study’s aim was to explore healthcare professionals’ perceptions of function-focused care education for the development of an education model using a shared mental model (SMM) in NHs. Semi-structured interviews with 30 interdisciplinary practitioners from four different professions (nurses, physical therapists, occupational therapists, and social workers) and focus group interviews with 12 experts were conducted. Data were analyzed using content analysis, and the education model development was guided by the shared mental models for data interpretation and formation. Our FFC interdisciplinary educational model incorporates four key learning components: learning contents, educational activities, educational goals/outcome, and environment, and four types of SMMs: team, task, team interaction, and equipment. As for educational contents, a team’s competencies with FFC were found to be team knowledge (physical and psychosocial functional care), team skills to perform FFC successfully (motivation, coaching and supporting, managing discomfort), and team attitude (possessing philosophy perceptions regarding FFC). As for learning outcomes, the shared cognition-based education model suggests not only the evaluation of practitioners, but also the assessment of residents’ aspects.

## 1. Introduction

### 1.1. Background: The Ageing Population and Nursing Homes in Korea

South Korea is one of the fastest aging countries in the world, having become an aged society in 2018 (the proportion of people over the age of 65 is 14.3%), and is expected to become a superaged society by 2025 to have the second highest ratio of aging population after Japan by 2050 [1,2]. In South Korea, long-term care insurance for the elderly has been implemented since 2008, providing long-term care for the elderly with major geriatric diseases and functional disabilities in care homes [3]. With a rapidly ageing population, the number of South Korean nursing homes (NHs) increased by about 270% from 1332 in 2008 to 3595 in 2019 and, during the same period, the number of elderly people admitted to NHs increased to 174,015 from 81,252, an increase of about 214% [4]. NH residents have various physical (e.g., impaired physical mobility, self-care deficit) and psychosocial (e.g., impaired memory, depression) care needs while presenting with multiple comorbidities (e.g., dementia, diabetes mellitus, arthritis) [5,6]. In recent years, more older patients with higher-level functional dependence have been admitted to NHs than in the past [7]. As the ageing population increases, more elderly people with low functional and cognitive abilities are expected to be hospitalized in Korean NHs, and ultimately, the number of residents who need functional care is expected to increase.

### 1.2. Function-Focused Care in Nursing Homes

Functional status means the basic level of activity an individual performs in order to fulfill the needs of daily life in relation to the various dimensions of life: the physical, psychological, psychosocial, and spiritual [8]. Functional decline has been reported to predict a low quality of life (QoL), poor physical health, repeated hospitalizations, and mortality [9]. In particular, the continuing functional assessment after admission to an NH home is considered to be a key measure of the quality of care provided [10]. Therefore, as functional care becomes more important, the concept of functional care has increasingly shifted from focusing on what caregivers do for elderly patients to function-focused care (FFC) that encourages patients to participate in their own health care. FFC, previously referred to as restorative care, is a care philosophy that evaluates the elderly’s remaining functional abilities and physical activity, provides interventions to optimize and maintain functional abilities, and increases physical activity time [11]. More recently, in a conceptual study on FFC in an NH setting, five assessment categories (physical condition, cognitive condition, changed function, ability to cooperate with caregivers, and motivation) and five intervention categories (plan to improve function through emotional stability, strengthening physical resources, stimulating motivation for self-care, repetitive musculoskeletal strengthening, and minimizing damage) were identified by practitioners [12]. Thus, FFC should be performed with the cooperation of NH multidisciplinary care staff for the purpose of helping the elderly use their remaining functions as safely as possible so that they can perform the functions for as long as possible.

### 1.3. Function-Focused Care Competency Development Need of Interdisciplinary Nursing Home Staff

There is sufficient evidence that FFC is a central aspect of successful functional caring. From a systematic review on FFC in various care settings, the results provided evidence supporting the efficacy of FFC (less functional decline or maintenance of function) and the safety of FFC (the number of falls did not increase significantly) [11]. In addition, a recent systematic review of FFC in NHs confirmed that FFC is beneficial to psychosocial functions (e.g., mood, affect, and behavioral problems), physical functions (e.g., activities of daily living, movement, and balance) and cognitive functions (e.g., visual memory, verbal memory, and room finding) [13]. Despite this evidence, it has been reported that nurses have a tendency to conceptualize their role as protecting elderly patients from adverse events and encouraging them to take part in activities that they consider to be risk-free for older adults [14,15]. Particularly in long-term care facilities, most older adults do not participate in a sufficient amount of physical activity [11], and it has been reported that the elderly’s cognitive functional status declined more after entering a nursing home [16].

In order to effectively implement FFC interventions in the elderly in NHs, a change in the perception of care practitioners is the first step, necessitating FFC education care practitioners. Since healthcare practitioners’ perceptions, such as a fear of the risks associated with physical activity, have been reported as important barriers to engaging older adults in physical activity [17,18,19], FFC education for healthcare practitioners is critical. Most studies to date [20,21,22,23,24,25,26,27,28,29,30,31,32,33] have provided some training to staff members in a facility or home setting as part of the FFC intervention. In addition, a review article evaluating the effectiveness of FFC identified that the contents of a FFC learning program included the assessment and evaluation of resident’ needs, physical activity, positioning, mobility, and transfers, ADLs, communication, residents’ emotions, and physiological feedback [13]. In accordance with these findings, although the direct care workers had learned motivational strategies and FFC intervention skills necessary for the elderly to perform their activities, the participation of multi-disciplinary direct care workers was insufficient, and the education was mainly focused on physical activity education.

In 2020, a study [34] investigating FFC training for interdisciplinary caregivers (social workers, physical therapists, and occupational therapists) was conducted in a Korean NH. In Korea, since the establishment of the long-term care insurance (LTCI) system in 2008, it is compulsorily that NHs are evaluated for their quality by the National Health Insurance (NHI) Corporation every three years, and the results are published on the LTCI website [35]. The quality evaluation index for the service provision process includes a comprehensive assessment of residents’ needs, establishment of a service provision plan, and evaluation of systematic case management by interdisciplinary service providers [36]. While the evidence for FFC interventions for NH settings is growing, there is little study for the FFC training of interdisciplinary staff, such as an education model. Thus, exploring how to educate the FFC for interdisciplinary practitioner to training in assessing and managing NH residents’ functions will help to promote high-quality functional care practices. With this study, therefore, we seek to explore effective implementation strategies of FFC education in an NH setting.

### 1.4. Analytical View and Theoretical Approach

In this study, the shared mental model (SMM) provided the framework for the interview guide and the interpretation of findings. Some healthcare experts have proposed that team training should focus more on a collective team process, such as a SMM [37,38]. The SMM is a widely used team training activity model that provides a similar cognition of a clinical situation, identified care plans, and work required of team members [34,39,40,41,42,43]. The SMM is a team-based approach in which team members possess information, such as team members’ skills, knowledge, behavioral tendencies, and attitudes, that enables them to perform better across different domains [44]. In a scoping review of health profession learners, Floren (2018) defined SMM as a cognitive expression of the task or/and team knowledge common to those who interact as a team to pursue a common goal for patient care [42]. The more the interdisciplinary healthcare approach of FFC is emphasized, the greater the need for an SMM-based functional care training strategy. However, we could not find any Korean study of interdisciplinary healthcare professions’ perspectives on FFC education in NHs. A real insight into the interdisciplinary FFC training strategy in NHs is the first step toward acquiring a solid understanding of the strategies of FFC education in Korea, as well as toward creating culturally appropriate FFC education tools and FFC programs for healthcare providers.

### 1.5. Research Objective

Effective interdisciplinary teamwork outcomes require a shared mental model framework that includes the team’s knowledge, skills, attitude, dynamics, and environment. We aimed to investigate how shared mental model-based FFC education can enhance the FFC competency of interdisciplinary practitioners within nursing homes. An additional goal was to describe the FFC education model available to NH healthcare providers.

## 2. Materials and Methods

This study utilized a qualitative exploratory design that included in-depth interviews and focus group interviews to explore the phenomenon [45]. This was a qualitative study of healthcare professionals’ perceptions of FFC education in Korean NHs that incorporated in-depth interviews (IDIs) and focus group discussions (FGDs). IDIs are a primary tool for collecting data about FFC education strategies using an SMM framework, and the FGDs method complemented the FFC education strategies uncovered during the IDIs.

### 2.1. Nursing Home Setting and Recruitment

The study took place in five NHs for older people with long-term care insurance—three public NHs run by government agencies and two private NHs run by individual nurses. The five NHs were located in Seoul and Gyeonggi-do and have 49 to 150 beds each. Different regions and organizations were investigated to accommodate the contextual diversity of Korean NHs. NH managers introduced us to interdisciplinary practitioners (we use this term inclusive of nurses, physical/occupational therapists, and social workers) who were directly involved in functional care and were considered to have received FFC training. Our interview participants came from these contacts.

### 2.2. In-Depth Interviews

A total of 30 in-depth interviews were held with practitioners who has worked more than one year in NH residents’ functional care. The participants were purposively selected to include interdisciplinary practitioners of NH residents with care levels 1 to 5 according to their mental and physical status. The inclusion criteria for the practitioners were that they needed to be working with interdisciplinary practitioners, to have been providing direct care to residents for at least one year in an NH, and to be willing to participate. We interviewed eighteen and twelve respondents at public and private nursing homes, respectively, until no new information was gained and saturation was achieved. The broad questions for exploring FFC education based on an FFC framework included the following:

(1) What specific knowledge do you need to manage the elderly’s functional status?
(2) Why do you think that knowledge is important and helps in functional management?
(3) How would you help and support other practitioners during functional care?
(4) What knowledge do you need from other practitioners during functional care?
(5) What are your beliefs and attitudes about functional care?

The interviews were conducted by trained researchers with experience in qualitative studies, lasted about 40 min each, and were audio recorded.

### 2.3. Focus Group Discussions

Two focus group discussions with six members each were conducted. Participants in the first-round focus group discussions included representative NH practice experts (two nurses, one social worker, one physical therapist, one occupational therapist, and one nutritionist), and the second-round focus group discussions included six multidisciplinary educators (five professors of physical therapy, occupational therapy, social welfare and nursing and one NH manager). Participants were purposively selected from four colleges and from among nursing homes previously interviewed in NHs. After formal introductions, the moderator briefed the participants on the study’s procedure and goals, as well as reporting the in-depth interview results before discussions commenced. Participants were given the following broad interview questions for discussion:

(1) What educational elements and content do you think should be shared?
(2) What teaching strategies can be applied, and which methods are effective?
(3) How should nursing home environmental factors be considered for FFC training?
(4) What do you think the educational goals for shared cognition-based FFC training should be?
(5) How do you think training should be evaluated?

A moderator facilitated the discussions, which each lasted about 60 min, while an assistant researcher audio-recorded the conversation and took field notes.

### 2.4. Data Analysis

Data from the IDIs and FGDs were transcribed verbatim by the first author, and the collected data were analyzed by both authors through content analysis using the SMM [46] as a guide for data interpretation and the development of a shared cognition-based FFC education strategy. The SMM identified four types of shared cognition: team, team interaction, technology (or equipment), and task models. Team models included teammates’ knowledge, skills, attitudes, preferences, and tendencies. Team interaction involved teammate-related aspects, and technology (or equipment) and task models comprised task context-related aspects. After reading the transcript data, meaning units were then coded, classified into categories, and organized into themes and subthemes [47] in order to identify, understand, and sort FFC education strategy into the four types of SMM characteristics.

### 2.5. Ethical Approval

The study was approved by the Korea University institutional review board(1040548-KU-IRB-16-57-A-3(R-A-1)). Participants participated voluntarily and were confident that the information they provided would be kept confidential. We obtained written informed consent from all IDI and FGD participants.

## 3. Results

The demographic characteristics of the IDI and FGD interviewees are summarized in Table 1. The population of IDIs consisted of 30 interdisciplinary practitioners from different fields (12 nurses, 8 physical (occupational) therapists, and 10 social workers). The average age of the participants of the interdisciplinary practitioner was 40.0 ± 9.12 years, the average total clinical experience of this practitioner group was 10.91 ± 6.29 years, and their elderly functional care experience was 5.64 ± 3.18 years.

The population of FGDs consisted of two interdisciplinary expert groups: NH practice expert group and multidisciplinary educator group. The average age of the NH practice expert group was 40.3 ± 13.0 years, the average total clinical experience of this practitioner group was 10.4 ± 7.5 years, and their elderly functional care experience was 5.0 ± 2.7 years. The average age of the educator group was 51.5 ± 5.3 years, their average total educational experience was 13.3 ± 7.5 years, and their total clinical experience was 14.2 ± 8.9 years.

**Figure 1:**
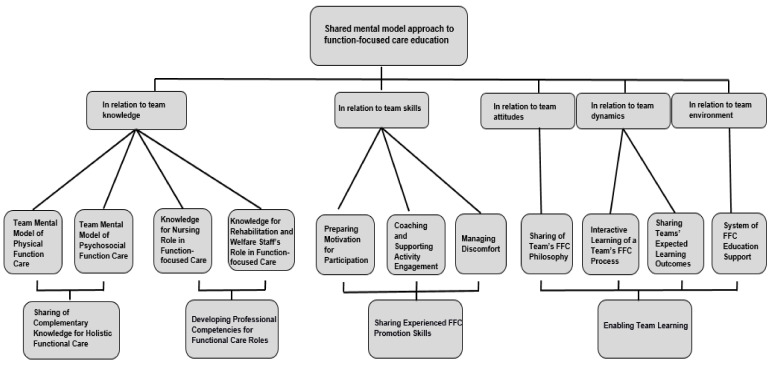
Coding tree for qualitative analysis.

The primary themes were (1) sharing of complementary knowledge for holistic functional care, (2) developing professional competencies for functional care roles, (3) sharing experienced FFC promotion skills, and (4) enabling team learning. We described function-focused education for nursing home practitioners with the help of subthemes and themes related to a shared mental model derived from the data. Figure 1 presents the subthemes and themes in the coding tree chart to illustrate the complexity of shared mental model-based FFC education.

### 3.1. Sharing of Complementary Knowledge for Holistic Functional Care

#### 3.1.1. Team Mental Model of Physical Function Care

One nurse said, “It is necessary to share knowledge about muscle strength and range of motion (ROM) exercises with a physical therapist in order to maximize the physical function of the elderly who use a walker” (Nurse 8; IDIs). Another nurse said, “I think it would be good to share how to exercise to maintain a resident’s status so that the elderly with Parkinson’s functions don’t deteriorate and whether it is better to walk with a walker than a cane” (Nurse 1; IDIs). The nurses emphasized gaining knowledge about mobility-related care (assessment and interventions) from physical therapists in order to care for residents’ physical function. Another nurse added, “I need to acquire information in regard to physical activity condition from the physical therapist about the patient’s ability to walk and how much daily exercise they should engage in” (Nurse 10; IDIs).

A physical therapist described the sharing of such knowledge: “For walking training for the elderly with Parkinson’s, it is first necessary to share the nurse’s knowledge of the resident’s basic health condition (e.g., symptoms and treatment). Besides, if we teach nurses how to help residents with Parkinson’s exercise, the nurses can help with exercise in the elderly’s daily care” (Physical therapist 1; IDIs). Another physical therapist said, “We need treatment knowledge about the resident’s disease from the nurse because we can change the physical therapy plan according to the patient’s health condition” (Physical therapist 5; IDIs). An occupational therapist described his view of sharing knowledge: “For occupational rehabilitation, we need a resident’s medical history, such as orthostatic hypotension or surgical history. Also, I can ask the social worker to provide a judgment about a resident’s condition on the reaction the patient had during the program activity” (occupational therapist 2; IDIs). One social worker described her perspective on sharing knowledge: “We can provide activity services tailored to the elderly’s Mini-Mental State Examination (MMSE) level. We also need an understanding of the resident’s major assessment and treatment (e.g., risk of falling, ADLs, ROM, disease, and medications). While I am not alone in caring for the patient, I think that customized care is possible only if I gain basic knowledge for functional care from the nurses’ or physical therapists’ specialized knowledge” (Social worker 3, IDIs). Another social worker said, “Since human functions work organically, we need to consider the physical functioning state of the elderly to provide a program that can give them a sense of psychological comfort, and then the psychosocial functions and relationship with the therapist will improve” (Social worker 8; IDIs).

#### 3.1.2. Team Mental Model of Psychosocial Function Care

Nurses also described the knowledge needed for psychosocial functional care. One nurse stated, “Since I don’t know much about various activity programs for the elderly, I need to acquire knowledge from a social worker about what the programs can accomplish, taking into account individual abilities and preferences. When older people sometimes do not move, this activity information is helpful in discriminating whether it is due to fatigue or depression” (Nurse 8; IDIs). One nurse added, “We need a social worker’s knowledge of a one-handed leisure program for paralyzed elderly people” (Nurse 10; IDIs). Another nurse stated, “I think it’s important to use an approach for overall function care. For the functional care of depressed elderly people, it would be good to exchange knowledge on providing religious activities or community-related services (e.g., conversation service, etc.) with a social worker. I think emotional function care will help to increase the satisfaction and self-confidence of the elderly in NH life and promote participation in activities” (Nurse 11; IDIs). Some nurses described cognitive function care: “From an occupational therapist, we learned about cognitive functional care programs (e.g., coloring in a pattern, matching word cards, etc.) for dementia residents with a bedridden functional state” (Nurse 2; IDIs).

### 3.2. Developing Professional Competencies for Functional Care Roles

#### 3.2.1. Knowledge for Nursing Role in Function-Focused Care

Nurses described nursing knowledge related to FFC as assessment knowledge of physical symptoms and signs that can quickly alert them to small functional changes (e.g., asymmetric facial expressions), as well as knowledge of the symptoms of the elderly’s disease (e.g., stroke, dementia). One nurse stated, “As the stroke progresses in the elderly, the weakening of one arm can be expressed as a pain in the arm. Since the elderly’s verbal expressions are inaccurate, we must be able to detect changes that differ from the existing state through physical assessment” (Nurse 3; IDIs). Another nurse described nursing knowledge of FFC as follows: “The older adults’ problem can be overlooked because the signs and symptoms in the elderly can be non-specific, such as indigestion or other health problems manifested by mild fever. Therefore, we must learn to assess the symptom-related causes of older adults more comprehensively” (Nurse 11; IDIs). Nurses also emphasized a knowledge of disease management, such as complications (e.g., urinary tract infections, pain, bedsores, and insomnia) related to QoL. One nurse said, “The residents in the facility are elderly people who take medicines for various diseases, so complications and exacerbations are very common. For example, urinary tract infections are especially common in older people who are lying down. Therefore, we need to know about managing complications, be able to educate staff to prevent them, and consult with doctors if necessary. Because older people frequently have urinary tract infections and decreased appetite and can also develop bedsores, we need to be aware of nursing knowledge about prevention and intervention for health problems” (Nurse 6; IDIs). Regarding work knowledge, the nurses mentioned not only knowledge of disease management, but also knowledge of psychosocial nursing or ADLs care. One nurse stated, “By evaluating depression for elderly people who are depressed, it is important to try to have a lot of conversation with them and give them emotional support during nursing care because it allows them to live the rest of their lives meaningfully. By periodically evaluating ADLs, we also frequently integrate exercises to prevent joint contraction into daily nursing care so that the elderly can live on their own as much as possible” (Nurse 3; IDIs).

#### 3.2.2. Knowledge for Rehabilitation and Welfare Staff’s Role in Function-Focused Care

The physical and occupational therapists who participated emphasized a knowledge of the gait and ADLs programs for their FFC work. One physical therapist stated, “If the elderly are able to walk independently, I plan for them to perform a fixed bike and gate exercise 3–4 times a week to increase their lower extremity muscle strength” (Physical therapist 2; IDIs). Another physical therapist stated, “I may plan functional care of 10 min of upper and lower joint exercise 4 times a week, using a knowledge of the musculoskeletal structure and ROM exercise therapy, while paying attention to prevent secondary damage caused by excessive joint exercise for the elderly” (Physical therapist 6; IDIs). Another physical therapist said, “If the elderly have suffered a stroke, it is necessary to use balance sense training to improve their walking” (Physical therapist 1; IDIs). One occupational therapist added, “Because elderly people with dementia or stroke or Parkinson’s have different conditions, ADL occupational therapy (e.g., eating with a spoon, dressing oneself) for hand tremors must be customized according to the health problem” (Occupational therapist 2; IDIs).

The social workers emphasized a knowledge of psychosocial intervention programs as their FFC work knowledge. “We need to be taught how to counsel for dealing with a patient’s emotional problems. Also, we must have knowledge of emotional support care programs that can be applied depending on the cognitive condition, such as elderly people with problem behaviors due to dementia, and resources (e.g., churches, community service organization) that can provide emotional support” (Social Worker 4; IDIs). The experts pointed out that NH healthcare providers have to be able to help with some work role. “Physical therapists maintain the gross function of the elderly, occupational therapists are in charge of detailed functions, and environmental factors and social background are managed by social workers. However, not all NHs staff all of these therapists enough, so it is important for facility managers to flexibly adjust some of the roles according to their actual workforce” (Professor of physical therapy; FGDs).

### 3.3. Sharing Experienced FFC Promotion Skills

#### 3.3.1. Preparing Motivation for Participation

NH practitioners emphasized the importance of helping staff motivate NH residents to engage in daily activities. Those skills include communication (e.g., giving praise), putting up good image posters, informing about and promoting positive functional activity outcomes, and matching residents with a group that is participating well together. One nurse stated, “We need to be taught how to motivate patients. Sometimes, when I praise an elderly patient for one action in bathing, the patient will be satisfied with the action and they will try the next action” (Nurse 1; IDIs). Nurses and social workers described communication skill as a valuable FFC skill: “It is difficult, but we need to constantly encourage patients to do activities. After the patient participates, if it feels like a good experience, the patient will participate well the next time” (Nurse 2; IDIs). Physical therapists and social workers described the need to provide interesting programs for motivation: “If the elderly do not want to be active alone, it is better to engage them in group activities. For example, if the elderly participate in group activities, such as a falls prevention physical activity, and see what others are doing, they can easily imitate the activity” (Physical therapist 1; IDIs). “If an elderly person refuses to participate in the program, it is recommended that they participate in a variety of programs composed of their favorite content. Changes in the emotional state of the elderly during the program should be observed” (Social Worker 4; IDIs).

#### 3.3.2. Coaching and Supporting Activity Engagement

Nursing home practitioners described the need for training skills in a step-by-step approach tailored to the patient’s functional level, with an emphasis on empathic attitude. This means not providing the elderly unconditional assistance, but rather engaging them in performing activities that require the least possible help. A key for accomplishing activity is the skill to use verbal or nonverbal cues to have residents try to complete an activity as best they can and to encourage them to do so alone. One nurse said, “When an elderly person eats, we may help them access step-by-step activities with specific instructions such as ‘It’s a spoon,’ ‘Pick it up,’ and ‘Chew it,’ rather than general instructions like ‘Eat’” (Nurse 2; IDIs). Another nurse added, “While caring for the elderly, if we continue to pay attention to the resident in a way that induces action, the elderly become interested in the activity. However, residents don’t do an activity well if we don’t care about the activity” (Nurse 3; IDIs). An occupational therapist stated, “We need a supporting skill to grasp the remaining functions of the elderly by body part and that are required according to the activity content. If the elderly resident can do the activity content, it is still necessary to guide and supervise the activity. But if the elderly’s remaining function makes the activity impossible, it will require auxiliary tools” (Occupational therapist 1; IDIs). One social worker stated, “When taking a bath, we induce the patient so that even if the patient does not try to do it well on his own, he can clean himself even where his arm touches” (Social worker 3; IDIs). Another social worker added, “We need to learn practical skills that can help the elderly be active by themselves” (Social worker 5; IDIs).

#### 3.3.3. Managing Discomfort

NH practitioners stated that discomfort can be a barrier to elderly residents participating in functional activities. They therefore described the need to learn skills that create a comfort zone when elderly patients faced unexpected situations, such as fear, fatigue, pain, or depression. One nurse said, “If a resident has a fear of falling when training to walk, it is necessary to eliminate that fear through explanation and support” (Nurse 10; IDIs). Another nurse said, “When a resident is anxious, we can take a walk together. Sometimes when the elderly resident is angry, we can offer relaxation therapy, such as a scented massage. At those times, we should approach the resident with an attitude of acceptance rather than criticism” (Nurse 2; IDIs). One physical therapist said, “I usually make a situation where the elderly can feel stable and emotionally comfortable before starting physical therapy” (Physical therapist 2; IDIs). One social worker stated, “When a resident does not wish to perform a functional activity, it is better to assess whether it is because of tiredness or depression” (SW 1; IDIs). Another social worker added, “At the time of a program activity, the elderly patient are frequently angry and uncooperative, so it is necessary to emotionally stabilize them” (SW 3; IDIs).

### 3.4. Enabling Team Learning

#### 3.4.1. Sharing of Team’s FFC Philosophy

This subtheme emphasized a shared attitude toward beliefs and values regarding FFC for empowering function care. Healthcare profession groups discussed the need for learning FFC philosophy. They also understood that shared attitudes and beliefs about FFC philosophy can influence team positivity, confidence, motivation, and affect effective interventions. One nursing expert said, “It is important to be taught common values and concepts in shared cognition-based function care. FFC philosophy sharing should be given priority because it generates a team’s motivation and attitude toward FFC education” (Professor of nursing; FGDs). A rehabilitation expert added, “Our facility basically shares the concept of FFC in the management of physical functions, so we are trying to support the activities that the elderly can do with their remaining abilities, without putting possible restrictions on the activities that the elderly want to do” (Occupational therapist; FGDs). A welfare expert offered, “Team spirit and a professional attitude is necessary for FFC education. As care practitioners, we need to learn FFC philosophy, such as function care goals and importance and partnerships in working” (Professor of social welfare; FGDs).

#### 3.4.2. Interactive Learning of a Team’s FFC Process

Teaching approaches for “interactive team learning” were discussed, not only formal education classes (e.g., online education) but also in-service-integrated learning (e.g., case management conferences, handover meetings, and interaction channels such as social network services). An important point discussed was that educational methods should be mutual. Through team interaction courses in education, practitioners can recognize their complementary roles under mutual goal setting and coordinate appropriate care service collaboratively. One nursing expert said, “Since multidisciplinary healthcare providers have different working hours, it will be more effective to simulate the care process using an online education platform rather than an offline one for education on shared cognition” (Professor of nursing 1; FGDs). Another nurse said, “When a caregiver performs functional care related to a fall during work, we can correct it immediately, so it is effective to do the fall prevention education together at work” (Nurse 3; IDIs). Another nurse offered, “In our facility, we learn functional care through case management meetings where multidisciplinary therapists come together to share treatment content and goals and to re-adjust care plans through evaluations” (Nurse 4; IDIs). A rehabilitation expert said, “If an elderly person refuses an exercise program because he or she is in a bad mood, the caregiver writes a report that day and shares it with other caregivers to help solve it” (Physical therapist; FGDs). A welfare expert explained, “Because interdisciplinary cooperation is important, caregivers can check with each other on how they are performing different aspects of care and consider developing a case-based education program that focuses on the individual functioning issues of residents” (Professor of social welfare; FGDs). A nursing expert said, “Simulating the FFC process, I think it would be best to have a problem-oriented approach sharing how to care for each case using case studies, role plays, and appropriate materials” (Professor of nursing 1; FGDs).

#### 3.4.3. System of FFC Education Support

The expert groups discussed the need for instructional and organizational development (e.g., education materials, nursing recording tools, physical rehabilitation apparatus, and facility policies) for supporting FFC education. One nurse noted, “Training opportunities such as online/offline refresher training related to FFC education are helpful” (nurse 1; FGDs). A rehabilitation expert said, “There are practical limitations in function care if the nursing home manager is passive when resources are needed for rehabilitation” (Physical therapist; FGDs). A welfare expert suggested, “If the facility has educational materials such as manuals, including ones on elderly-specific care and FFC interventions, it will be helpful in the actual work situation” (Social Worker; FGDs).

#### 3.4.4. Sharing Teams’ Expected Learning Outcomes

There was discussion about caregiver’s short-term outcomes that increased their educational satisfaction, positive FFC intervention attitudes and perceptions, improvement of knowledge and skills, and increasing FFC behavior. In addition, participants discussed NH elderly residents’ long-term outcomes that have a positive effect on residents and their families’ satisfaction, residents’ functional status (e.g., increase in functional activity) and QoL. They viewed caregivers’ outcomes to be to encourage residents to engage in activities, take satisfaction in their care, and to optimize a healthy, functional life. The expert groups also described outcome evaluations and feedback as means of promoting education.

A nursing expert said, “In terms of care providers, it is important outcome that they enhance their knowledge and skills and practice FFC to improve the elderly’s quality of life” (Nursing professor; FGDs). A rehabilitation expert added, “In the long run, I think that both the practitioner and the elderly should take a satisfaction survey. This training will also need to assess how well the understanding of the FFC has improved” (Occupational therapist; FGDs). Another rehabilitation expert stated, “The outcomes may be evaluated in the long term as to whether the caregiver’s shared cognition has increased the functional activity and improved the functional status of the elderly according to the FFC” (Physical therapist; FGDs). A welfare professional suggested, “When we manage the functional problems of the elderly, I think it is good to evaluate how the attitude to care has changed before and after the training” (Professor of social welfare; FGDs).

## 4. Discussion

**Figure 2:**
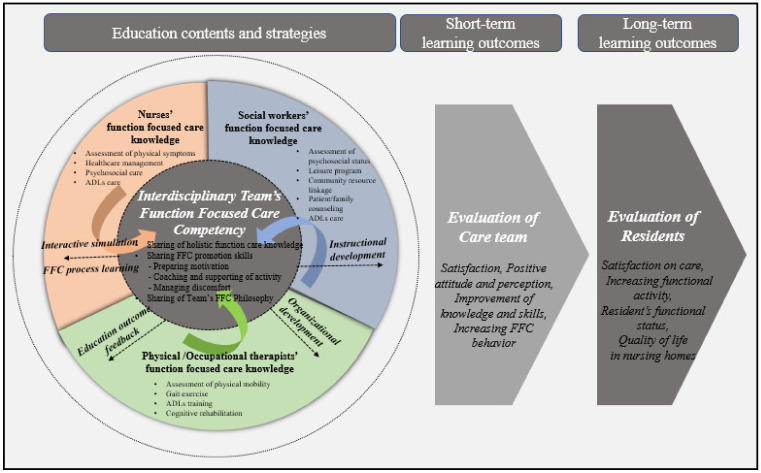
Preliminary FFC education model for interdisciplinary practitioners in nursing homes.

One important finding of the study is that we propose a function-focused care education model for multidisciplinary healthcare provider teams in NHs (Figure 2), the first such model proposed in South Korea. To clarify the basic configuration of FFC training, the results of in-depth interviews and focus group discussions were synthesized with the conceptual framework of the SMM. The three shared cognition components (team’s complementary knowledge, team’s skill, and team’s philosophy) in the inner circle characterizes caregiver teams’ competency with FFC, and the three components in the next circle are each caregiver’s work knowledge (nursing knowledge, social welfare knowledge, and physical/occupational therapy knowledge) related to function-focused care. The arrows around the outer circle indicate educational strategies in the context of facilities that affect team competency improvement. The educational evaluation, corresponding to the educational goals, characterizes elements in two aspects: caregiver and resident. If the FFC ability of caregivers is first improved through education, it is expected that the function and QOL of the elderly in the facility will improve as well.

The SMM is a widely used team training activity model that provides a similar cognition of a clinical situation, identified care plans, and works required of team members [39]. Mathieu et al. (2000) [46] adapted Cannon-Bowers et al.’s SMM to present four types of SMMs: team, job/task, team interaction, and equipment/technology. Discussion of the results of this study according to this shared mental model type follow below.

First, team members share teammates’ knowledge, skills, and attitudes related to the SMM of function care. This content could be confirmed by educational contents. Consistent with previous studies, FFC education included the following knowledge: (1) the FFC philosophy of breaking residents’ dependencies during ADLs, (2) skills such as communication (verbal cues, motor cues, verbal prompts, verbal encouragement, etc.) or physiological feedback (management pain, fear, fatigue, etc.), and (3) assessment, care goal setting, and intervention (physical activity, ADLs). Although the existing research investigated FFC education concerning ADLs care intervention mainly for nursing staff, this study presented a team training approach to shared team knowledge of FFC for effective holistic function care. It was confirmed that the contents of the psychosocial function management (e.g., depression) and physical/occupational function management (e.g., fall prevention training) were added to the interdisciplinary team knowledge.

Second, team members must have shared task knowledge about how to perform duties related to the SMM of function care. This content could be confirmed by the evaluation of educational outcomes. Most of the existing studies on FFC were based on intervention rather than on education, and the outcomes focused in part on residents’ activity or mood [11]. However, overall positive learning outcomes are achieved when caregivers consistently apply FFC behaviors to attain resident’s function care goals, which also results in the elderly participating more voluntarily in FFC with a greater level of satisfaction. A study of systematic reviews showed that professional development activities focused on health-centered education are associated with positive effects on participants’ satisfaction, knowledge, skills, attitudes, and behavioral change [48]. In addition, a study by Lee et al. [13] showed improved educational outcomes (transactive memory system, knowledge sharing and utilization, team outcomes, motivation for learning transfer, self-efficacy, and interpersonal understanding) in indicators related to shared cognition in interprofessional function care training. In SMM, the integration of the multidisciplinary knowledge of the healthcare providers is a precondition for effective team performance and teamwork [49]. Thus, a team’s shared mental outcomes influence the active application of interdisciplinary teams’ knowledge and skills in NHs. In Korea, elderly people with a disability grade I (requires help in all aspects of ADL) to V (dementia with limited functional decline) can be admitted to NHs, and in principle, elderly people who need assistance with almost all daily activities can enter NHs [35]. Therefore, in regard to educational outcomes, it may take a long period of time to see the results of improving their function, and function maintenance can be regarded as function improvement. In this study, educational outcomes were divided into long-term and short-term results based on expert opinions reflecting this context.

Third, team members must have a common understanding of how teams interact related to the SMM of function care. This content could be confirmed by the training method of team’s interactive learning. Regarding FFC’s teaching methodology, Lee’s (2019) systematic review study [13] on FFC confirmed that interactive training such as role-plays, workshops, and case studies were conducted to elicit feedback from instructors. In this study, which focuses on team education, an education program that demonstrates the care process as a way to discuss with each other during work or to share the care contents of other occupations was proposed. An NH is a complex system in which different occupations work in different roles [50,51]. Interdisciplinary approaches are interactive, with health care professionals adapting their specific care goals to common goals, coordinating interventions through interprofessional team meetings and decision-making within a multilateral interaction process [52,53]. Simulation is recognized as an important tool to facilitate the development of SMMs and has been frequently used in team training in intensive care units [38,54]. In particular, simulations promote a better understanding and support of team members and enable developing an awareness of the value of shared cognition in achieving goals [37]. In Korea, offline case meetings are held regularly by multidisciplinary teams according to national facility evaluation criteria; therefore, if functional care case simulations are developed as part of online education, it will be accessible to all caregivers. Furthermore, our results strongly suggest that interprofessional teams provided a way to train the elderly with specific functional problems following the FFC process.

Last, team members need to understand the equipment normally used because the control of such equipment is especially important for effective team functioning. This content could be confirmed by the NHs’ educational environment. If the facility’s organizational/instructional resources (supportive learning environment, education materials, etc.) are adequately developed to support employee training on functional care, the capacity of its caregivers can be strengthened through team training activities. Function-focused care can only be successfully achieved by merging the resources of the NH with its team’s knowledge. However, we have found that the difficulties faced by caregivers in accessing necessary educational resources (e.g., a lack of practical guidelines) have been reported as barriers in the team environment. Further studies are required to develop education resources such as function care guidelines for continuing interprofessional team training and the implementation of FFC.

This interdisciplinary FFC education model is intended to crystallize an understanding of the relationships of educational components within the SMM framework. Because an NH is a complex health care system, sharing the team’s SMM on FFC analyzed in this study is important for interdisciplinary practitioners’ cooperative activities. A SMM was also confirmed in a recent qualitative study conducted with a team on geriatric care in a hospital setting [40]. In this study, participants confirmed the importance of teamwork, such as shared mental models of tasks. In Korean NHs, interdisciplinary practitioners are already actively participating in the functional assessment and management of the elderly [12]. Nevertheless, although much nursing knowledge about functional care has been accumulated, there have been few studies on FFC education for interdisciplinary practitioners. Therefore, this study is meaningful in that it presents interdisciplinary’ FFC education model for holistic functional care that has been studied in various NH contexts and can be applied in practice.

The generalizability of the study’s interdisciplinary education model is limited because the study included only a small sample of participants (30 practitioners and 12 experts) from just 5 NHs in some regions near the Seoul city. Therefore, this may limit the transferability of the findings to other older patient care settings, such as rural or small private NHs and hospitals. Nonetheless, this model was developed and agreed in collaboration with interdisciplinary practitioners in representative NH settings: nursing and rehabilitation professionals, including those from social welfare services and the NH management sector. The proposed FFC education model advocates strategies for implementing a care philosophy of FFC that promotes the functional ability of residents in NHs through an endeavor to improve practitioners’ shared cognition and a sense of worth to those contributing in team care. Based on our findings, further studies are needed to assess the effectiveness of a continuing interdisciplinary education program in NHs built on this preliminary education model. It will also be necessary to test this educational model and how its factors impact learning outcomes with a larger sample across more NH facility settings for greater time lengths, with both practitioners and residents. In addition, applying interdisciplinary education model for NH practitioners’ FFC delivery, an advanced FFC education training can address several levels of functional decline.

## 5. Conclusions

The healthcare professionals’ perceptions of FFC education provides a means to empower and conceptualize team competency by showing the need of a team’s shared cognition that is feasible in the NH context to provide effective instruction. This study illustrates a novel FFC education model, inductively developed from the accounts of interdisciplinary healthcare professionals (nurses, physical therapists, occupational therapists, and social workers) from community NHs. This model enacts the philosophical nursing underpinnings of FFC, developing them into a lived synthesis of shared mental models put into NH practice, a process which transformed the teams’ understanding of learning. Taking into account the functional status that an individual achieves, along with his or her varying levels of capabilities (physical, cognitive, psychological, and social), the dynamic team FFC intervention process is inevitably important in care effectiveness. Implementation of the shared cognition-based FFC education model could enable integrated team functional care for the elderly with various physical and psychosocial health problems, its delivery guided by the elderly’s healthcare needs. Tackling interdisciplinary FFC education in this way could achieve the community residents’ highest level of functionality.

## Figures and Tables

**Table 1:** Characteristics of the in-depth interview and focus group participants.

Variables	Category	In-Depth Interview -Interdisciplinary Practitioners (*n* = 30)	Focus Group 1 -NH Practice Experts (*n* = 6)	Focus Group 2 -Multidisciplinary Educators (*n* = 6)
		Mean (±SD) or *n* (%)	Mean (±SD) or *n* (%)	
Age (yrs)		40 (9.12)	40.3 (13.0)	51.5 (5.3)
Gender	Male	3 (10)	-	1 (16.7)
	Female	27 (90)	6 (100)	5 (83.3)
Education	Associate’s degree	12 (40)	3 (50)	-
	Bachelor’s degree	8 (27)	3 (50)	-
	Master’s degree	10 (33)	-	1 (16.7)
	Doctoral degree	-	-	5 (83.3)
Occupation	Nurse	12 (40)	2 (33.3)	-
	Nurse manager	-	-	1 (16.6)
	Physical (or occupational) therapist	8 (27)	2 (33.3)	-
	Social worker	10 (33)	1 (16.6)	-
	Nutritionist		1 (16.6)	-
	Professor of nursing	-	-	2 (33.3)
	Professor of physical therapy, occupational therapy	-	-	2 (33.3)
	Professor of social welfare	-	-	1 (16.6)
Working years in elderly functional care		5.64 (3.18)	5.0 (2.7)	-
Education career		-	-	13.3 (7.5)
Length of time as practitioners		10.91 (6.29)	10.4 (7.5)	14.2 (8.9)

SD = Standard Deviation; Percentages (%) are based on the total number of participants in each group (*n*).

## Data Availability

Data is available upon request from the corresponding authors.
